# Control of Membrane Fusion Mechanism by Lipid Composition: Predictions from Ensemble Molecular Dynamics

**DOI:** 10.1371/journal.pcbi.0030220

**Published:** 2007-11-16

**Authors:** Peter M Kasson, Vijay S Pande

**Affiliations:** Department of Chemistry, Stanford University, Stanford, California, United States of America; University of California San Diego, United States of America

## Abstract

Membrane fusion is critical to biological processes such as viral infection, endocrine hormone secretion, and neurotransmission, yet the precise mechanistic details of the fusion process remain unknown. Current experimental and computational model systems approximate the complex physiological membrane environment for fusion using one or a few protein and lipid species. Here, we report results of a computational model system for fusion in which the ratio of lipid components was systematically varied, using thousands of simulations of up to a microsecond in length to predict the effects of lipid composition on both fusion kinetics and mechanism. In our simulations, increased phosphatidylcholine content in vesicles causes increased activation energies for formation of the initial stalk-like intermediate for fusion and of hemifusion intermediates, in accordance with previous continuum-mechanics theoretical treatments. We also use our large simulation dataset to quantitatively compare the mechanism by which vesicles fuse at different lipid compositions, showing a significant difference in fusion kinetics and mechanism at different compositions simulated. As physiological membranes have different compositions in the inner and outer leaflets, we examine the effect of such asymmetry, as well as the effect of membrane curvature on fusion. These predicted effects of lipid composition on fusion mechanism both underscore the way in which experimental model system construction may affect the observed mechanism of fusion and illustrate a potential mechanism for cellular regulation of the fusion process by altering membrane composition.

## Author Summary

Membrane fusion is the transport process used for neurotransmitter release, insulin secretion, and infection by enveloped viruses. The precise mechanism of fusion is not yet understood, nor is the means by which membrane properties such as composition and curvature affect the fusion process. Here, we use molecular-dynamics simulations of lipid vesicle fusion under different lipid compositions to generate a more detailed explanation for how composition controls membrane fusion. We predict that lipid composition affects both the initial process of forming a contact “stalk” between two vesicles and the formation of a metastable “hemifused” intermediate. These two roles act in concert to change both the rate of fusion and the level of detectable fusion intermediates. We also present initial results on fusion of vesicles at different membrane curvatures. Recent experimental results suggest that the creation of highly curved membranes is important to fusion of synaptic vesicles. Our simulations cover a curvature regime similar to these experimental systems. In combination with previous results, we predict that the effect of lipid composition on fusion is general across different membrane curvatures, but that the rate of fusion is controlled by both composition and curvature.

## Introduction

Membrane fusion plays a key role in cellular function, allowing processes as diverse as neurotransmitter release, secretion of peptide hormones, and infection by enveloped viruses. Fusion is also critical in allowing intracellular transport. The cellular membranes participating in these fusion reactions contain complex mixtures of lipids and proteins. The composition and potentially the organization of these mixtures are both variable and closely regulated by the cell [1–4].

A variety of experimental and computational model systems [5–14] have been used to gain insight into the basic physical properties underlying membrane fusion. Such model systems are of necessity much simpler in nature than the physiologic context for fusion, containing one or a few lipid species and omitting or simplifying the protein environment. In designing and interpreting model systems for fusion, it is important to consider how variations in the lipid mixtures chosen may affect the results. Investigating how changes in lipid composition affect the kinetics and mechanism of fusion also provides fundamental insight by illuminating which aspects of fusion are robust to small perturbations to the model system used and which are highly model-dependent.

Experimental data on lipid vesicle fusion indicate that variation in lipid composition plays an important role in determining fusogenicity. Much initial work concentrated on the effects on fusion by lipids that prefer either positive or negative membrane curvature [5,15]. Perhaps most extensively characterized is the association between increased fusogenicity and increased fraction of phosphatidylethanolamine (PE) [7], a lipid headgroup thought to promote negative membrane curvature. Plasma membranes in fusogenic contexts (synaptic vesicles, synaptic membranes, viral membranes) have a ratio of phosphatidylcholine (PC) to PE headgroups measured at between 0.9 and 2.0 [16–18]. The presence of PE has been identified as a critical factor for fusion [19–21], and many systems used to develop structural models for fusion intermediates contain a substantially higher proportion of PE than physiologic levels [3,8,22]. Similarly, theoretical treatments of proposed fusion intermediates suggest that PE is important to the energetic favorability of the stalk-like state that has been proposed as an early fusion intermediate [8,10,23–25]. Other lipid species likely play an additional modulatory role in fusion, and the PC:PE ratio in physiological membranes also varies independently in the inner and outer leaflets [26,27]. In this work, we examine the effect of PC:PE ratio as a first-order approximation of lipid composition effects; we first treat uniform composition in the inner and outer membrane leaflets and then examine asymmetric compositions.

Membrane curvature has long been believed an important modulator of the fusion process. Simply conceived, the curvature strain of a highly curved membrane can provide a driving force that reduces the energetic barrier for the fusion process. Most physiological fusion occurs between membranes that have a relatively large radius of curvature (e.g., synaptic vesicles, virions, cells) or are even concave relative to the fusion site (presynaptic terminals, endosomes). However, the local curvature at the site of fusion has been a subject of much discussion, with several theories positing a membrane protrusion with high local curvature at the site of fusion [28–32]. Recent experimental evidence suggests that synaptotagmin, one of the neuronal fusion proteins, preferentially binds to curved membranes, induces tubulation of model membranes with a diameter of 17.5 nm, and accelerates vesicle fusion rates [33].

This important finding provides more direct evidence that highly curved membrane structures such as we simulate here may play an important role in physiological fusion and that induction of curvature may be a major function of the fusion protein apparatus. The possibility that physiological fusion may involve highly curved membranes raises the questions of to what degree this curvature is required for fusion and how curvature may differentially affect the formation and stability of fusion intermediates. Here, we report initial results in that regard; we refer the reader to the forthcoming work by Lee and Schick for a more extensive investigation of this phenomenon from a field-theoretic perspective (Lee and Schick, unpublished data).

Because the structures and detailed kinetics of fusion intermediates are difficult to observe experimentally, computational simulations of fusion play an important role in generating detailed structural and kinetic models for fusion that are consistent with available experimental data. Coarse-grained simulations (such as the early rigid-amphiphile work by Noguchi and Takasu [34]) allow a substantial increase in computational tractability at the expense of some fidelity to experiment; the degree of fidelity varies with the particular approximations made in constructing the model. The lipid model developed by Marrink and Mark [35] provides a quantitative approximation of experimentally observable lipid parameters such as area per head group, diffusion, and bilayer width, and represents a good compromise between computational tractability and level of atomistic detail. Initial results reported for membrane fusion with this model [11] demonstrate the fusogenicity of 15 nm vesicles composed of either pure palmitoyloleoyl-PE (POPE) or a dipalmitoyl-PC:dipalmitoyl-PE (DPPC:DPPE) mixture, also observing faster fusion pore formation in the DPPC:DPPE mixture than in pure POPE (*n* = 6 and *n* = 4 simulations, respectively). Simulated pure DPPC vesicles were not observed to form fusion pores.

**Figure 1:**
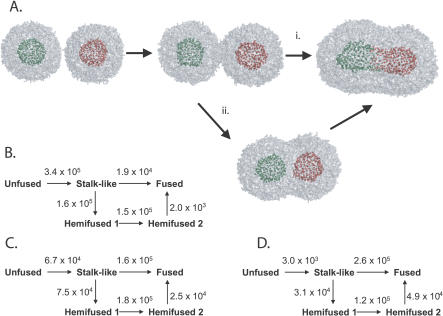
Reaction Rates and Mechanisms for Vesicle Fusion (A) Representative structures from unfused vesicles and each major fusion intermediate: unfused vesicles, a stalk-like state, a hemifused intermediate, and fully fused vesicles. Vesicles fuse either via a direct pathway from stalk-like to fully fused (i) or via an indirect pathway (ii) using a metastable hemifused intermediate. Surfaces rendered in gray represent solvent-accessible area; red and green spheres denote the inner-leaflet phosphate groups of each vesicle respectively. (B–D) Reaction rates in s^−1^ determined for each reaction pathway at each lipid composition. (B) Rate for pure POPE. (C) Rate for for 1:1 POPC:POPE. (D) Rate for for 2:1 POPC:POPE. Hemifused 1 and Hemifused 2 denote structurally similar late-hemifused states; kinetic differentiation of these states was determined by kinetic consistency analysis (see Methods). Hemifusion occupies a sufficiently large area of state space that inverconversion between some hemifused microstates is slow, resulting in kinetic differentiation.

In subsequent work, we have used this Marrink-Mark coarse-grained model to systematically predict intermediates and kinetics for the fusion of small POPE vesicles [36]. Our simulations suggested a branching fusion pathway in which vesicles first react to form a stalk-like intermediate but then either rapidly form a fusion pore from the stalk-like state (the “direct” pathway) or fuse via an intermediate with an expanded hemifusion diaphragm that is metastable on the microsecond timescale (the “indirect” pathway; see Figure 1 for a structural illustration). Previous theoretical work (reviewed in [37]) has suggested these two pathways (without kinetic detail) as alternative hypotheses; we have predicted that these pathways may coexist within a single system. In our initial report, we predicted pure POPE vesicles to react via both pathways but to primarily undergo fusion via the late-hemifused intermediate using the indirect pathway. We now extend this model to address how more complex mixtures of lipids may affect the reaction mechanism and kinetics of fusion.

Physiologic membrane fusion takes place in membranes consisting of a complex [3,16–18] and likely inhomogeneous [38–42] mixture of multiple lipid species and proteins. Experimental and computational model systems for fusion are far simpler, containing well-defined lipid mixtures; in the case of computational studies, single-component membranes are routinely used [10,12,43]. Given this gap in complexity between model systems used for mechanistic studies of fusion and the physiologic situation they are meant to reproduce, we wish to understand how fusion mechanisms and properties depend on membrane composition. We have therefore performed thousands of simulations of vesicle fusion at different lipid compositions to systematically predict how membrane fusion kinetics and intermediates depend on lipid composition. This molecular-dynamics approach yields similar results to those recently obtained using self-consistent field theory methods [44]. Our molecular-dynamics analyses, the field-theoretic results of Schick and coworkers, and prior continuum-mechanics work by Kozlov expand computational models to more complex membrane compositions to better approximate experimental models and physiologic scenarios.

## Results

**Figure 2:**
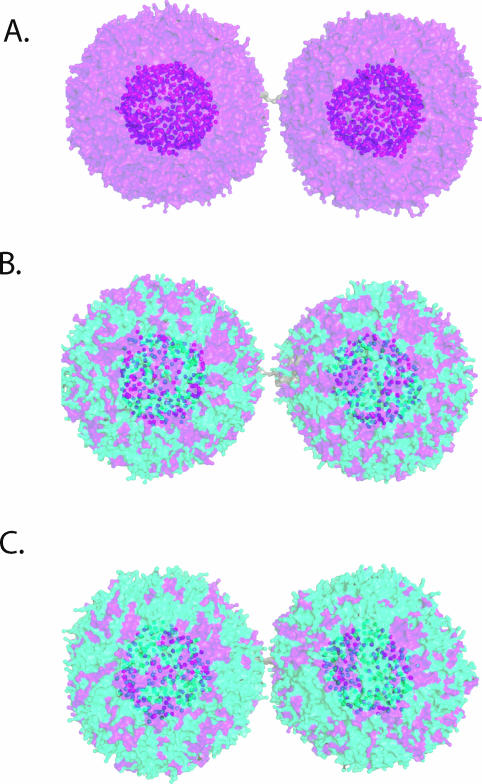
Starting Structures for Vesicles at Three Different Lipid Compositions (A) Pure POPE vesicles. (B) Vesicles composed of a 1:1 POPC:POPE mixture. (C) Vesicles composed of a 2:1 POPC:POPE mixture. POPE lipids are rendered in pink, POPC in purple. Rendered surfaces represent the solvent-accessible surface of the vesicle; spheres represent the phosphate groups of the vesicle inner leaflet lipids. Vesicles are linked by a single crosslinker molecule.

To predict the effect of lipid composition on membrane fusion mechanisms, we have performed massively distributed molecular-dynamics simulations of vesicle fusion at different lipid molar ratios of palmitoyloleoyl-PC (POPC) and POPE. Starting conformations for the 15 nm vesicles used are displayed in Figure 2. The resulting dataset contains ≥1,000 separate simulations of vesicle fusion, up to 1 ms in length, at each lipid composition. We have used this dataset to construct kinetic models for fusion and to assess how the kinetics, mechanisms, and intermediates of fusion vary with increasing PC:PE ratio. We show that the same basic pathways underlie fusion reactions in our simulations at all compositions studied. However, our simulation data also suggest that the relative frequency of reaction pathways and relative stability of reaction intermediates vary substantially. Increased PC content in vesicles is associated with increased utilization of the direct fusion pathway, with the result that different lipid compositions have different preferred reaction pathways for fusion. We also report results on how membrane curvature affects rates of fusion stalk formation in our simulations; while fusion proceeds more slowly at lesser curvatures, the effect of lipid composition appears to operate independently of vesicle curvature.

### Kinetics and Mechanism of Fusion at Different Lipid Compositions

**Figure 3:**
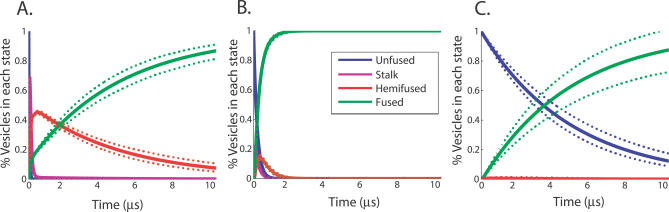
Kinetics of Vesicle Fusion Simulated at Different Lipid Compositions The relative concentrations of fusion intermediates and fused vesicles are plotted for each time point of the simulation for (A) pure POPE, (B) a 1:1 POPC:POPE mixture, and (C) a 2:1 POPC:POPE mixture. Predictions are derived from MSM analysis of all fusion trajectories. Dotted lines represent 90% CIs.

We use Markovian state models (MSMs) to analyze the kinetics and mechanism of fusion in a systematic manner. Described in detail in previous work on both protein folding and membrane fusion [36,45–48], MSMs provide a means for systematically analyzing large numbers of molecular-dynamics trajectories to yield quantitative predictions of membrane fusion mechanisms. By constructing a series of MSMs at different lipid compositions, we are able to predict the long-timescale kinetics for vesicle fusion at each lipid composition (Figure 3). We use measurements of lipid and contents mixing between vesicles to characterize each structural snapshot as unfused, stalk-like (outer leaflets fusing with a small contact area), hemifused (outer leaflets fusing with a large contact area), or fully fused (Figure 1A; for details, see [36]). We have recently introduced a topological metric for assessing fusion intermediate structures: persistent voids [49]. For the fusion of 15 nm vesicles, the width of the hemifusion “neck” between the outer leaflets of each vesicle correlates relatively well with the extent of lipid mixing. Model-building and analysis of molecular-dynamics data were performed both independently for each lipid composition and in a conjoint manner (see Methods), yielding similar rates and mechanisms via each method (see Figure S1 for a detailed comparison).

The kinetic profiles for fusion predicted via MSM analysis (Figure 3) show a number of differences in the kinetics of fusion intermediates as vesicle composition varies from pure POPE through increasing concentrations of POPC. When the vesicle composition is changed from pure POPE to 1:1 POPE:POPC, more closely approximating the average physiologic ratio in cell membranes, the unfused state decays approximately 5-fold more slowly (*t*_1/2_ of 36 ns for POPE versus 184 ns for POPC). However, the fused state is formed almost an order of magnitude more quickly (*t*_1/2_ of 4,212 versus 348 ns, respectively). This difference results in part from altered usage of the stable late-hemifused intermediate.

When the fraction of POPC is increased to 67% (2:1 POPC:POPE), this trend continues further. The unfused state decays with a *t*_1/2_ of 4.8 μs, and the timescale for fusion is dominated by the slow reaction of the unfused vesicles. We predict a decrease in fusogenicity from a 1:1 PC:PE ratio to a 2:1 PC:PE ratio (rates given below; see Table S2 for an energetic analysis) that is in qualitative agreement with experimental studies of PEG-induced vesicle fusion [7]. The overall rate of formation for fused vesicles containing 2:1 POPC:POPE approximates that of pure POPE (*t*_1/2_ 4.2 versus 4.8 μs using a single-exponential approximation), although the mechanism of fusion differs substantially between these compositions in our simulations.

**Figure 4:**
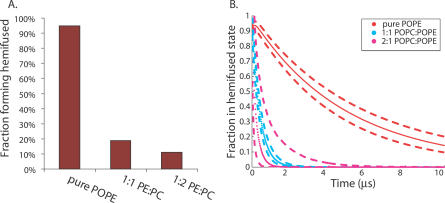
POPC Content Associated with Decreased Formation and Decreased Stability of the Hemifused State (A) The fraction of all trajectories that form a hemifused state is plotted for each vesicle composition. Vesicles with greater POPC content are less likely to form hemifused states. (B) The time-evolution of the hemifused state is plotted for each vesicle composition. In vesicles with greater POPC content, the hemifused state is less stable and decays more quickly to form fully fused vesicles. Dashed lines represent 90% CIs. Because vesicles containing a 2:1 ratio of POPC:POPE rarely form hemifused intermediates, the sampling uncertainty for the reaction of hemifused vesicles is proportionately greater.

Much of this mechanistic difference derives from energetic changes in the late-hemifused pathway. Considered as an ensemble, vesicle fusion reactions at higher POPC:POPE ratios have a lower concentration of late-hemifused intermediates than do fusion reactions of pure POPE vesicles. This results from both a decreased usage of the hemifused pathway (Figure 1A, pathway ii; Figure 4A), leading to fewer hemifused intermediates formed, and decreased stability of the hemifused state (Figure 4B). Although 95% of pure POPE vesicle fusion trajectories form hemifused intermediates, only 19% of trajectories containing a 1:1 POPC:POPE mixture and 11% of vesicles containing a 2:1 POPC:POPE mixture form hemifused intermediates. The remainder of fusion trajectories transition rapidly from stalk-like states to fused states without isolated expansion of a hemifusion diaphragm.

Our previous analyses have shown that such rapid fusion from the stalk-like state involves concerted conformational change in both inner and outer vesicle leaflets. Using our MSM analysis of fusion trajectories, we predict *t*_1/2_ values of 5,320, 424, and 123 ns for the decay of the hemifused state in pure POPE, 1:1 POPC:POPE, and 2:1 POPC:POPE vesicles, respectively (decay plots shown in Figure 4B). We postulate that the decreased usage of the hemifusion pathway and decreased stability of hemifused vesicles results from increased free energy of the hemifused state relative to the fused state.

We have also computed detailed predictions of rates and mechanism at each lipid composition using our MSM analysis (Figure 1 and Table 1). These data show that rates of stalk formation decrease with increasing POPC concentration in the simulation (*p* < 0.001, pairwise Kolmogorov-Smirnov tests), in accordance with previous theoretical predictions [10,25]. The reaction rate from stalk-like to metastable hemifused states decreases with increasing POPC concentration (*p* < 0.001), and the rate of fusion pore formation by hemifused states increases significantly with the introduction of POPC (*p* < 0.001). The rate of direct fusion pore formation from the stalk-like state also increases significantly (*p* < 0.001).

**Table 1:**
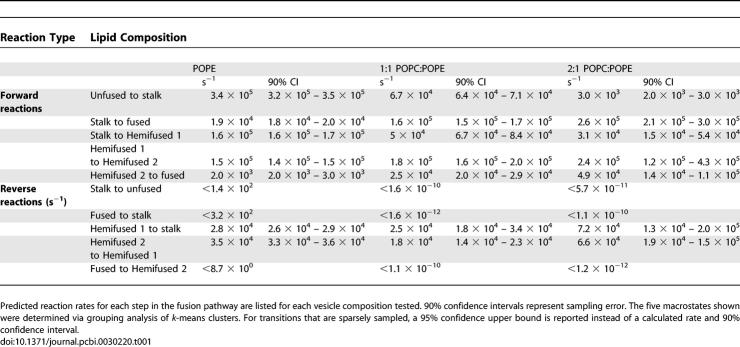
Predicted Reaction Rates for Fusion

### Analysis of Transition State Energies

**Figure 5:**
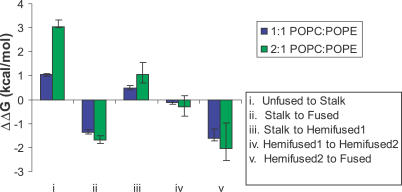
Variation of Transition State Energy with Lipid Composition Plotted are ΔΔG^‡^ values for transition state energies calculated with respect to fusion of pure POPE vesicles. Transition state energies are determined as specified in Table S2, and error bars represent 99% CIs determined via nonparametric analysis.

Using classical transition state theory, one can interpret these rate changes in terms of free energy of activation using a single-barrier approximation. Such a model oversimplifies the large coordinated rearrangements involved in fusion; nonetheless, it allows gross prediction of how transition state energies vary with lipid composition. Relative free energy values for each transition state at each lipid composition are listed in Table S1. Analysis of the difference in free energies for each transition state (Figure 5) shows significant differences in activation free energies as a function of lipid composition. As these data are derived from the rate calculations, the trends are similar, but analysis in terms of activation free energies eases comparison between reactions and between lipid compositions.

We predict large (≥1 kcal/mol) and significant ΔΔG^‡^ values for the following reactions when pure POPE vesicles are exchanged for a 1:1 POPC:POPE mixture: formation of the stalk-like state from unfused vesicles (Figure 5, reaction i), formation of the fused state from the stalk-like state (Figure 5, reaction ii), and formation of the fused state from the hemifused state (Figure 5, reaction v). The activation energy for formation of the hemifused state from the stalk-like state also increases significantly (*p* < 0.001, Kolmogorov-Smirnov test), but the magnitude of the change is smaller. Interestingly, when the POPC:POPE ratio is further increased to 2:1, the largest energy change we calculate is for the intial reaction of unfused vesicles to form a stalk-like state. Relative to fusion of pure POPE vesicles, the ΔG^‡^ for formation of a stalk-like state increases by 1.0 kcal/mol when the lipid composition is changed to 1:1 POPC:POPE and by an additional 2.0 kcal/mol when the composition is changed to 2:1 POPC:POPE.

### Local Lipid Concentration in Mixed POPC:POPE Vesicles

Since POPE is considered a more fusogenic lipid than POPC, we examined whether fusion in POPC:POPE mixtures was associated with a local increase in POPE concentration at the site of stalk formation. Such local lipid concentration differences are believed important to cellular fission in cytokinesis, as immobilization of PE lipids leads to incomplete cell division [21]. We observe a mean-squared lipid displacement of 26 nm^2^ (compared with a vesicle surface area of 170 nm^2^) from the simulation start to formation of the stalk-like state, allowing for a moderate degree of lipid mixing over the vesicle surface. This degree of motion allows for good local mixing of lipids within each leaflet prior to fusion but is likely not sufficient to observe any long-timescale ordering or lipid phase separation that may occur. After fusion, our simulations display extensive mixing of lipids between corresponding leaflets of each vesicle.

In this context, we observe no change in relative density of lipids in the region of stalk formation for any lipid composition tested (Figure S2), although vesicles undergo fusion at all compositions. Our simulations are not designed to detect slow-timescale positional ordering of lipid mixtures, as we sample a single starting state (albeit 1,000 times) for each lipid concentration in docked vesicles. Experimental evidence suggests that hydrocarbon tail groups (identical between POPC and POPE) are a determining factor in the ordering behavior of lipids [50,51], and previous simulations have suggested that PC/PE mixtures do not phase-separate in bilayers [52]. We therefore predict that a 2:1 ratio of POPC:POPE is sufficiently fusogenic not to require local lipid reordering for fusion, although we cannot rule out slow-timescale ordering of lipids, and our simulations are not designed to test the effects of lipid phase-separation or similar behavior.

Membrane tension is another means by which the energetic favorability of fusion can be altered. As membrane composition can also affect tension, this is one potential mechanism for the composition effects we predict. Our analysis assumes that the coarse-grained force field used accurately reproduces relative differences in membrane tension between lipid compositions.

### Independent Variation of Inner and Outer Leaflets

**Figure 6:**
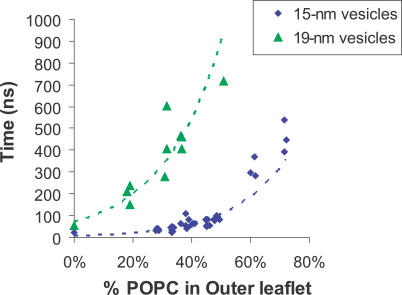
Dependence of Stalk Formation on Fraction POPC in Outer Leaflet Plotted are average times for reaction of unfused vesicles to initiate a hemifusion stalk, as determined by analysis of simulations with differing inner and outer leaflet compositions. Stalk formation times are plotted for both 15 nm vesicles (diamonds) and 19 nm vesicles (triangles); single-exponential fits to the data are shown (*r* ^2^ = 0.88). Linear fits to the data yield *r* ^2^ values of 0.67 (0.79 with the 0% POPC datapoint omitted) and 0.81, respectively. Initial stalk formation is assessed here by mixing of >10 lipids between vesicle outer leaflets.

We also simulated vesicle fusion over a range of asymmetric POPC:POPE compositions in which we varied the POPC content in the outer and inner leaflets independently. Such asymmetry is prominent in physiologic bilayers [26,27,53] and has been found to influence fusogenicity in experimental model systems [54,55]. A total of 4,500 individual simulations were performed to test 30 such compositions, with POPC content in each leaflet ranging from approximately 10% to approximately 50%. Over this range, we predict that the formation of an initial stalk-like state from the unfused state depends strongly on outer leaflet PC:PE ratio (Figure 6; *r* ^2^ = 0.79, *p* < 0.0001) but does not depend significantly on inner leaflet PC:PE ratio. Lee and Schick predict a similar dependence using self-consistent field theory [44]. After accounting for the influence of outer leaflet composition on stalk-like state formation rate, no further correlation was detected with either inner leaflet composition or total PC:PE ratio (residuals from a linear fit of outer leaflet composition to stalk-like state formation were uncorrelated with either of these variables). Rates of fusion (assessed as mean estimated time to fusion) displayed a more complex dependence on lipid composition (Figure S3 and Table S2). These rates were weakly correlated with inner leaflet composition (*r* ^2^ = −0.52, 95% CI −0.78 to −0.17, *p* = .006) but displayed a nonlinear dependence on both inner and outer leaflet compositions.

### Dependence on Membrane Curvature

To probe the effect of membrane curvature on vesicle fusion, we have simulated fusion under two additional curvature regimes: fusion of two somewhat larger 19 nm diameter vesicles and fusion of a 15 nm diameter vesicle to a planar bilayer. In each case, the membranes were apposed at 1 nm separation and linked by a chemical crosslinker in a manner identical to the simulations of 15 nm vesicle fusion.

#### Vesicle-bilayer fusion.

In our simulations of 15 nm vesicles tethered to planar bilayers, we did not observe either stalk formation or fusion over 400 simulations at 1:1 POPC:POPE of up to 940 ns in length using a bilayer patch of size 25 nm × 25 nm, an additional two simulations of 5 and 8 μs in length, respectively, or six simulations using a bilayer patch of 50 nm × 50 nm and ranging from 8 to 20 μs in length. If one treats the formation of a stalk-like structure as Markovian on the microsecond timescale or faster, these trajectories can be combined to estimate an upper bound on the reaction rate. Using a Poisson analysis, we estimate the 90% confidence upper bound to be 5,710 s^−1^ for the reaction of a 15 nm vesicle with a 50 nm × 50 nm bilayer patch to form a stable stalk-like structure (see Protocol S1 for a derivation). Curvature fluctuations in the planar bilayer almost certainly affect the rate of fusion. It is well known that simulations with small periodic boundaries can artificially damp such fluctuations [56,57]; although we have tried to minimize this effect by using a large 50 nm × 50 nm patch, such effects may still play a role in slowing the rate of fusion. The effects of bilayer curvature on membrane fusion will be the subject of future work; Schick and coworkers also address bilayer-vesicle fusion in a forthcoming paper (Lee and Schick, unpublished data).

#### Fusion of 19 nm vesicles.

To assess the generality of the lipid-composition effects we observe on fusion, we have simulated the fusion of 19 nm vesicles. A total of 11 lipid compositions were tested, varying the inner and outer leaflets independently between 0% and 50% POPC. Four simulations of 2 μs each were performed at each composition; kinetics of each reaction are plotted in Figure S4. We report initial results on how fusion rates depend on membrane curvature here; a more systematic characterization will be presented in future work. Rates of stalk formation between vesicles are plotted in Figure 6 as a function of outer leaflet POPC composition. We observe a similar dependence on POPC fraction in both 15 nm and 19 nm vesicles; stalk formation is consistently slower in 19 nm vesicles than in 15 nm vesicles. In both cases, the time to stalk formation is strongly correlated with the fraction of POPC in the vesicle outer leaflet (*r* ^2^ = 0.81, *p* = .001 for 19 nm vesicles; *r* ^2^ = 0.79, *p* < .0001 for 15 nm vesicles). However, the relationship between stalk formation kinetics and fraction POPC is better fit by a single-exponential curve (*r* ^2^ = 0.88 and 0.82, respectively), which suggests a linear relationship between fraction POPC and ΔΔG of stalk formation.

Based on this exponential fit, we calculate a ΔΔG for initial stalk formation equal to 4.3 kT/mol fraction POPC in the outer leaflet (95% CI 3.6 to 5.1) for 15 nm vesicles and equal to 5.0 kT/mol fraction POPC for 19 nm vesicles (95% CI 3.6 to 6.4). Due to sampling considerations, we estimate the uncertainties on these energies at approximately a factor of two. In their recent manuscript on lipid composition effects on the fusion of two planar bilayers, Lee and Schick calculate energies for stalk formation between bilayers as a function of the stalk radius [44]. We use the values they report for bilayers of symmetric composition at a stalk radius of ∼1 nm to construct a quantitative link between their field-theoretic calculations and our molecular-dynamics simulations. A linear fit to their data yields a ΔΔG of approximately 10.7 kT/vol fraction POPC. This agrees with our predictions for 19 nm vesicles of asymmetric and symmetric composition to within approximately 2-fold. The molecular volumes of POPC and POPE differ by 6% [58,59]. We can then extrapolate this linear fit to a ΔG of zero to calculate an approximate Arrhenius factor for our fusion rates. We estimate the resulting Arrhenius factor at between 7.6 and 36 ns for 15 nm vesicles and between 63 and 150 ns for 19 nm vesicles; we emphasize that due to the uncertainties and extrapolations involved, this factor should be treated as accurate within approximately one order of magnitude (compared with the factor of two error predicted above for the energies; rates scale with *e* ^ΔG/kT^). Although this calculation involves a number of uncertainties, it represents to our knowledge the first direct quantitative link between continuum-theory and molecular-dynamics predictions for membrane fusion.

## Discussion

We have performed tens of thousands of molecular-dynamics simulations of vesicle fusion in order to predict the effects of different lipid compositions on fusion mechanisms. Systematic analysis of these simulations, up to 1 μs in length, shows the same underlying reaction pathways at all lipid compositions tested. However, the usage of these pathways differs significantly with lipid composition. Specifically, in our simulations, 95% of pure POPE vesicles fuse via a metastable hemifused intermediate in our simulations, while only 11% of 2:1 POPC:POPE vesicles form a metastable hemifused state, instead fusing rapidly from the stalk-like state without formation of an isolated hemifusion diaphragm. Recent experiments by Weisshaar and coworkers in an experimental model for synaptic vesicle fusion have yielded results that are in good qualitative agreement with our predictions: increasing the fraction of PE lipids in either fusion partner results in an increased frequency of experimentally detectable hemifused intermediates [60]. The reduced usage of hemifusion at lipid mixtures more closely approximating physiologic lipid compositions raises the possibility that isolated expansion of the hemifusion diaphragm may not be a major on-pathway structural intermediate of physiologic membrane fusion, and may instead represent a small population or an off-pathway intermediate that may reversibly undergo “reactivation” for fusion.

Our predictions therefore have several important consequences for experimental models of physiological fusion environments. We predict substantial changes to fusion mechanisms from changes in simple POPC:POPE mixtures that are still substantially less complex than the physiologic context for fusion. More complex lipid mixtures, the activity of fusion proteins, and the addition of pharmacologic agents to manipulate fusion may alter either local lipid composition or fusion reaction pathways. It is therefore possible that cellular fusion assays using mutant fusion proteins [61–63] or using pharmacologic perturbation [64] to slow fusion may observe different structural intermediates or reaction pathways than predominate in native physiologic fusion.

These complexities highlight the utility of analyzing fusion mechanisms and intermediates in multiple membrane environments, as it is unknown which lipid composition best approximates the combination of complex lipid–protein mixtures and fusion–protein activity that mediates fusion in living organisms. Vesicle size is an additional factor; how the size of the contact area in the stalk-like versus the hemifused state varies with vesicle size is as yet unresolved, as is the larger question of how vesicle curvature affects fusion mechanism. Our predictions of how lipid composition affects fusion kinetics and mechanism also illustrate the way in which cellular control of lipid composition may provide a means to regulate fusion. Immobilization of PE has been shown to inhibit cytokinesis in cellular fission assays [21]; we speculate that similar regulatory effects exist for fusion.

Our simulation analyses also highlight another important aspect of complex kinetic systems as applied to membrane fusion: multiple reaction mechanisms and multiple sets of reaction rates within a mechanism can lead to similar or even indistinguishable kinetics of product formation. This fundamental property of kinetic systems containing intermediates has been demonstrated via both experimental and theoretical analysis in the case of peptide–major histocompatibility complex protein dissociation kinetics [65,66]. In the context of membrane fusion, we predict that pure POPE and 2:1 POPC:POPE mixtures form fully fused vesicles and undergo vesicle contents mixing at very similar rates. However, a significant majority of vesicles fuse via different mechanisms. In this case, the two mechanisms could be distinguished via ultrafast measurements of lipid mixing and monitoring of unfused state decay, but this finding illustrates the caution that should be exercised in using reaction kinetics to establish mechanism without direct observation of reaction intermediates.

The influence of lipid composition on membrane fusion intermediates has received an extensive theoretical treatment from a continuum-mechanics perspective [10,23–25,29,32,67]. Our simulations provide an alternative approach by using molecular dynamics to treat lipid bilayers as discrete rather than continuous entities. This discrete approach is particularly advantageous in areas of high local curvature [25], and we have applied it to 15 nm vesicles that more closely resemble the ∼20 nm vesicles used by Lentz and coworkers [6,7] than the planar bilayers in which much of the continuum-mechanics theory has been developed [23–25]. The degree of local curvature in physiologic fusion may vary with cellular context, although some experimental evidence suggests that fusion occurs between highly curved membrane structures [68].

Our simulations agree qualitatively with continuum-theory approaches in how relative PC:PE content modulates the energetics of both the stalk-like [25] and the hemifused [23] states (here we refer to the isolated expansion of the hemifusion diaphragm). Our models use highly curved crosslinked vesicles as a starting state, which accounts for the decrease in energy required for formation of the stalk-like state. The incorporation of dynamic information is another important difference in our models, and it is the analysis of these molecular-dynamics trajectories that leads us to predict that small vesicles may fuse via two distinct pathways [36]: a “direct” pathway in which a fusion pore is formed from a stalk-like state via a coordinated change involving both inner and outer leaflets; and the canonical “indirect” pathway in which isolated expansion of the hemifusion diaphragm precedes pore expansion. Based on our calculations, we predict that energetic modulation of the direct pathway plays an important role in the control of vesicle fusion by lipid composition.

Our simulations of membrane fusion at different lipid compositions demonstrate the power of ensemble molecular dynamics to develop and refine models based on experimental data. By systematically analyzing multiple reaction conditions, we predict how simple model systems generalize to more complex scenarios and how multiple reaction mechanisms may be consistent with a single set of kinetic observations. Detailed kinetic analyses also enable the use of transition state theory to predict how changes in lipid composition modulate energetic barriers to the formation of fusion intermediates. All these analyses are enabled by our use of distributed computing to obtain thousands of molecular-dynamics trajectories, thus achieving robust statistical sampling. Sampling of this nature is particularly important for the analysis of complex systems such as membrane fusion, where multiple reaction pathways, multiple timescales, and a complex physiologic environment complicate the investigation of reaction mechanism and kinetics.

## Methods

### Molecular-dynamics simulations.

Simulations of POPE vesicles were performed as previously described [36]. Vesicles of 15 nm diameter, obtained via simulating the coalescence of lipid vesicles from an aqueous homogenate [69], were positionally restrained using a crosslinker of 1 nm in length between the amine groups of one lipid from each vesicle and apposed at a 1 nm minimum inter-amine distance approximating a “docked” conformation (Figure 2A). We have previously investigated the effects of varying the crosslinker length and initial separation distance [36]; here, we treat those effects as approximately independent of effects due to lipid composition. Lipids are represented using the Marrink-Mark model [35]; previous studies on this model have validated a number of bilayer structural and dynamic parameters against experimental values.

Vesicles containing a mixture of POPC and POPE lipids were created from the POPE starting structure by converting the amine to a tri-methylamine group for every second lipid (for a 1:1 mixture) or two of every three lipids (for a 2:1 mixture). Simulations were run in explicit solvent at 325 K with full electrostatic and Van der Waals interactions using the Marrink-Mark coarse-grained force field [35] and a version of GROMACS [70,71] adapted for the Folding@Home distributed computing architecture. A 4-fold timescale normalization was applied to the coarse-grained simulation trajectories as in the original parameterization and previous reports. Shifted potentials with a 1.2 nm cutoff were used for both Coulombic and van der Waals interactions, as in the original parameterization [35]. The Folding@Home project [72] was used to run ≥1,000 separate simulations for each lipid composition of up to 1,120 ns in length.

### Simulation of 19 nm vesicles.

The fusion of two 15 nm vesicles yields a larger vesicle; one of these fusion products was used as the basis for 19 nm vesicle simulations. Two copies of the vesicle were placed in a water box approximately 48 × 38 × 22 nm in size. These vesicles were placed 1 nm apart and linked by a 1 nm crosslinker as per the 15 nm vesicle simulations. The 19 nm vesicles were almost perfectly spherical; axes from the best-fit ellipsoid to outer leaflet phosphate coordinates were 93.4 A, 91.4 A, and 93.1 A, respectively. Simulations were performed using the same conditions as 15 nm vesicle fusion; at least four simulations of at least 2 μs in length were performed for each reaction condition tested.

### MSM analysis.

Structural snapshots were taken at 20 ns intervals from each simulation and analyzed for lipid and contents mixing as previously reported [36]. A reduced-dimensional space was generated for each simulation by assigning an ordered triple (inner, outer, contents) to each structural snapshot, where inner, outer, and contents are the number of molecules of each type that have mixed between the simulated pair of vesicles at the time of the snapshot. Initial grouping of snapshots was performed via *k*-means clustering in this reduced-dimensional space, using *k* = 8. An initial MSM analysis was performed on these clusters as described below. The clusters were then merged into macrostates according to the following kinetics consistency criterion: that time-propagation of merged system remains within error of the time-propagation behavior of the original system. Two clustering methods were used to analyze the simulation data: coclustering snapshots from all lipid compositions to produce a single set of cluster definitions, and then building independent transition matrices for each composition and performing all analysis independently for each composition. Coclustering facilitates precise comparison of kinetics and energetics across reaction conditions; see Figure S1 for a detailed comparison of results from coclustered and independent analyses. From conjoint analysis of all fusion trajectories, five macrostates were determined necessary to reproduce the time-propagation behavior of the model: these states were retrospectively characterized as unfused, stalk-like, fused, and two hemifused states named Hemifused 1 and Hemifused 2.

MSM analysis was performed as previously described [46] by constructing a transition probability matrix *A* such that each *a_ij_* is the frequency that a sampled trajectory in state *i* at time *t* will be in state *j* at time *t* + 20 ns. Given some starting distribution of states *R* at time *t*, the distribution of states at time *t* + *τ* will be *R* × *A* ^(*τ/20 ns*)^. Similarly, the rate matrix *K* for the system of macrostates is log(A) / 20 ns. Estimation of sampling error was performed by Bayesian resampling of transition count matrices using the Dirichlet distribution as previously described [45]. Hemifused state decay kinetics were calculated for each lipid composition by examining all molecular-dynamics trajectories to determine the ensemble of clusters corresponding to the first snapshot classified as hemifused in each trajectory, and then using MSM propagation analysis to calculate the decay of this ensemble. MSM analysis yields extremely low statistical sampling error; however, we estimate the potential systematic error for our kinetic analyses to be within 2-fold.

### Transition state energies.

Free energy values for transition states in the fusion reaction pathway were calculated at each lipid composition by approximating each reaction between fusion intermediates as having a single free-energy barrier and using the following relationship from transition state theory:

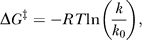
where *k* is the forward rate constant derived from MSM analysis, *R* is the gas constant, and *k*_0_ is the Arrhenius prefactor. Analysis of sampling error was performed by resampling from the transition count matrix according to the Dirichlet distribution.

### Radial distribution functions.

Radial distribution functions were calculated for each structural snapshot by taking the point of closest approach between lipids of each vesicle as the reference point and determining radial distribution projected onto a cylinder with a long axis measuring 6 nm oriented along the vector connecting the vesicle centers of mass. Because the vesicles are curved, a uniform distribution of lipid per unit surface area will have a nonuniform radial distribution when measured as a function of cylinder radius. Radial distribution functions for starting structures are therefore calculated as reference values.

## Supporting Information

Figure S1 Comparison of Kinetics between Coclustered and Separately Clustered Data Plotted in (A–C) are kinetics of vesicle fusion for pure POPE, 1:1 POPE:POPC, and 2:1 POPC:POPE lipids predicted via coclustering. In this procedure, MSMs are generated by coclustering all trajectory microstates across all lipid compositions to determine macrostates. The transitions between macrostates and the remainder of the model analysis are then performed separately on each lipid composition dataset. The advantage of coclustering is that macrostates are directly comparable between lipid compositions. Plotted for comparision in (D–F) are kinetics of vesicle fusion derived by clustering data and constructing an MSM independently for each lipid composition. Fusion kinetics are similar in both cases; the major difference is in the clustering boundary between stalk-like and hemifused for the pure POPE simulation; this difference gives rise to an approximately 2-fold difference in estimated *t*_1/2_ for formation of the fused state and an approximately 1.5-fold difference in estimated *t*_1/2_ for decay of the hemifused state (corresponding to a change in ΔG^‡^ of 0.26 kcal/mol). In general, although our statistical errors are extremely low, we estimate a systematic error in rate measurements of approximately 2-fold due to factors such as this.
(238 KB PDF)

Figure S2 Radial Distribution Functions for POPC and POPE Lipids Plotted are average radial distributions relative to the stalk contact point for first trajectory snapshot after stalk formation for each simulation run calculated for 1:1 POPC:POPE (A) and 2:1 POPC:POPE (B) vesicles. Because the vesicle surface curvature results in a nonuniform radial distribution profile, distributions from the start state are plotted for reference. Distributions plotted represent the average values over all molecular-dynamics trajectories. No relative enrichment of either POPC or POPE is observed at either lipid mixture.
(118 KB PDF)

Figure S3 Estimated Reaction Kinetics for Fusion at Asymmetric Lipid Compositions Differing between the Inner and Outer Vesicle Leaflets Vesicle fusion was simulated at 30 lipid compositions ranging between approximately 50% and approximately 90% POPC content in the inner and outer leaflets. Each plot shows the estimated time-evolution of one such set of fusion reactions, with time plotted on the *x*-axis and the fraction vesicles in each state plotted on the *y*-axis. Vesicle compositions are marked A–DD, corresponding to the compositions listed in Table S2. Because long timescales were not sampled as efficiently in these simulations, compositions where the fusion reaction proceeds slowly have higher error in the long-timescale kinetics predicted.
(205 KB PDF)

Figure S4 Kinetics of 19 nm Vesicle Fusion Plots show number of lipids mixed as a function of time. In each panel, the upper curve represents outer leaflet lipid mixing and the lower curve represents inner leaflet lipid mixing. Lipid mixing is averaged across all simulations at each lipid composition and vesicle size.
(2.4 MB EPS)

Protocol S1 Supplementary Methods (104 KB PDF)

Table S1 Relative Transition State Energies Energies are given in kcal/mol and calculated using forward rates obtained via MSM analysis of molecular-dynamics trajectories at each lipid composition using the relation ΔΔG^‡^ = −*k_B_*T ln *k* − ΔG_0_, approximating each reaction as a single-barrier energy landscape with ΔG_0_ set to the transition state free energy from unfused to stalk in pure POPE vesicles (*). 90% confidence intervals are generated via Dirichlet resampling of the MSM transition matrix. For reference, *k_B_*T = 0.645 kcal/mol.
(100 KB PDF)

Table S2 Asymmetric Lipid Composition and Times of Fusion Inner and outer leaflet molar ratios of POPC:POPE are listed for the 30 vesicle compositions plotted in Figure S3. Labels A–DD correspond to labels in the figure. Also shown are *t*_1/2_ values for reaction of unfused vesicles to form a stalk-like state and estimated mean times to full fusion for each composition. Mean times to fusion were estimated by calculating mean first passage times from the unfused to the fused state using a MSM constructed for each reaction condition. As noted earlier, the uncertainty in these estimates is higher because they reflect long-timescale behavior that is less well-sampled in the simulations. Values marked ** are too poorly sampled to estimate. Values marked * are estimated but with high uncertainty.
(130 KB PDF)

## Abbreviations

MSM: Markovian state model
PC: phosphatidylcholine
PE: phosphatidylethanolamine
POPC: palmitoyloleoyl-PC
POPE: palmitoyloleoyl-PE
