# Supplementary material for: Control of Membrane Fusion Mechanism by Lipid Composition: Predictions from Ensemble Molecular Dynamics
Source: PLoS Comput Biol. 2007 Nov 16;3(11):e220. doi: 10.1371/journal.pcbi.0030220 (PMC2077900; doi:10.1371/journal.pcbi.0030220)
Supplement: Supplementary file 1 [file pcbi.0030220.sd001.pdf]

Supplementary Methods:

Simulation of vesicle fusion with asymmetric lipid compositions

Fusion of vesicles with asymmetric inner and outer leaflet composition was simulated at 30 different compositions. For each composition, 20 starting states were created as follows, using a pure POPE vesicle starting structure. POPC:POPE molar ratios were selected for the inner and outer leaflets independently, and POPE lipids were randomly replaced with POPC across the surface of each leaflet until the desired molar ratio was achieved. The fusion of two such vesicles using a chemical crosslinker was simulated as described in the Methods. Five independent simulations were performed for each starting states, yielding a total of 100 simulations per composition. Structural snapshots were saved from each simulation at 20 ns intervals. To allow consistent comparisons, Markovian State Model (MSM) analysis was performed by assigning each structural snapshot to the clusters defined previously by k-means analysis and constructing a separate MSM for each composition.

Prediction of an upper bound on the rate of fusion between 15-nm vesicles and lipid bilayers

If we assume that fusion is a Markovian process on the microsecond timescale, we can effectively aggregate the simulations and assess stalk formation (the first stage of fusion) as a continuous-time stochastic process sampled on one long interval.

We can approximate this sampling via a Poisson distribution, in which the probability of observing n events during the time period of duration t is:

$$P(N(t)=n)=\frac{e^{-k\cdot t}(k\cdot t)^{n}}{n!}, \tag{S1}$$

where k is the rate of the underlying process.

Since we observe n=0 events during our simulation interval, we obtain:

$$P(N(t)=0\mid k)=e^{-k\cdot t} \tag{S2}$$

And via Bayes' rule:

$$P(k\mid n=0)=\frac{P(n=0\mid k)P(k)}{P(n=0)} \tag{S3}$$

$$P(k\mid n=0)=\frac{e^{-k\cdot t}P(k)}{\int_{k=0}^{\infty}P(n=0\mid k)P(k)dk} \tag{S4}$$

If we take the prior P(k) to be constant over all k, we obtain the probability density function:

$$P(k \mid n=0) = \frac{e^{-k \cdot t}}{\int_{k=0}^{\infty} e^{-k \cdot t} dk} = t \cdot e^{-k \cdot t} \tag{S5}$$

We integrate to obtain a 90% confidence bound for observing n = 0 events:

$$P(k > k_{upper}) = \int_{k=k_{upper}}^{\infty} t \cdot e^{-k \cdot t} = e^{-k_{upper} \cdot t} \tag{S6}$$

We have 100.8 us aggregate simulation time for each of 4 vesicles, so t = 401.2 μs. Letting P($k > k_{upper}$) = 0.10, we obtain k_{upper} = 5710 s^{-1}
