# Supplementary material for: Control of Membrane Fusion Mechanism by Lipid Composition: Predictions from Ensemble Molecular Dynamics
Source: PLoS Comput Biol. 2007 Nov 16;3(11):e220. doi: 10.1371/journal.pcbi.0030220 (PMC2077900; doi:10.1371/journal.pcbi.0030220)
Supplement: Supplementary file 2 [file pcbi.0030220.sg001.pdf]

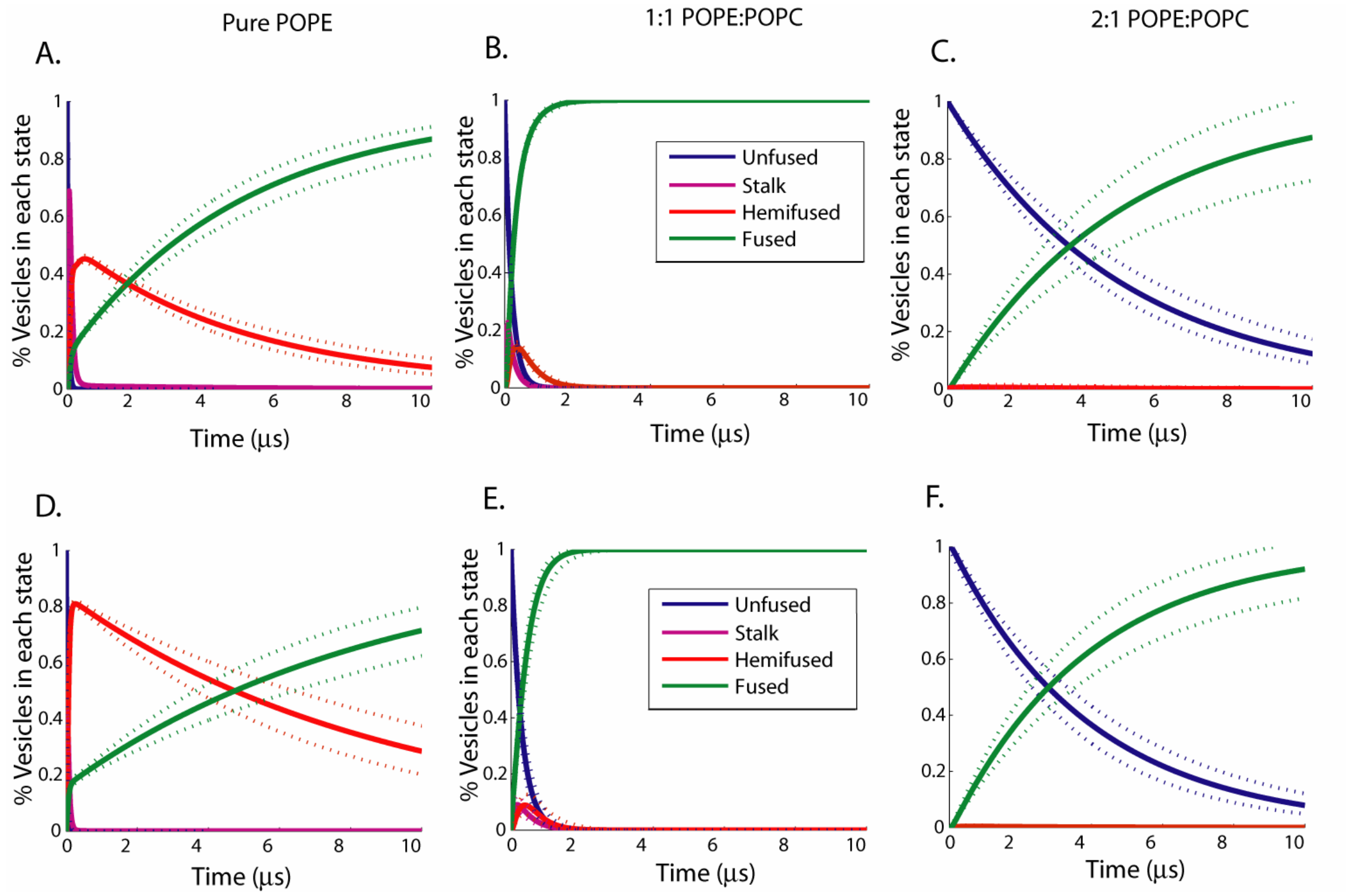

Figure S1. Comparison of kinetics between co-clustered and separately clustered data. Plotted in (**A-C**) are kinetics of vesicle fusion for pure POPE, 1:1 POPE:POPC, and 2:1 POPC:POPE lipids predicted via co-clustering. In this procedure, Markovian State Models are generated by co-clustering all trajectory microstates across all lipid compositions to determine macrostates. The transitions between macrostates and the remainder of the model analysis are then performed separately on each lipid composition dataset. The advantage of co-clustering is that macrostates are directly comparable between lipid compositions. Plotted for comparision in (**D-F**) are kinetics of vesicle fusion derived by clustering data and constructing an MSM independently for each lipid composition. Fusion kinetics are similar in both cases; the major difference is in the clustering boundary between stalk-like and hemifused for the pure POPE simulation; this difference gives rise to an approximately twofold difference in estimated $t_{½}$ for formation of the fused state and an approximately 1.5-fold difference in estimated $t_{½}$ for decay of the hemifused state (corresponding to a change in $\Delta G^{\ddagger}$ of 0.26 kcal/mol). In general, although our statistical errors are extremely low, we estimate a systematic error in rate measurements of approximately twofold due to factors such as this.
