# Supplementary material for: Control of Membrane Fusion Mechanism by Lipid Composition: Predictions from Ensemble Molecular Dynamics
Source: PLoS Comput Biol. 2007 Nov 16;3(11):e220. doi: 10.1371/journal.pcbi.0030220 (PMC2077900; doi:10.1371/journal.pcbi.0030220)
Supplement: Supplementary file 3 [file pcbi.0030220.sg002.pdf]

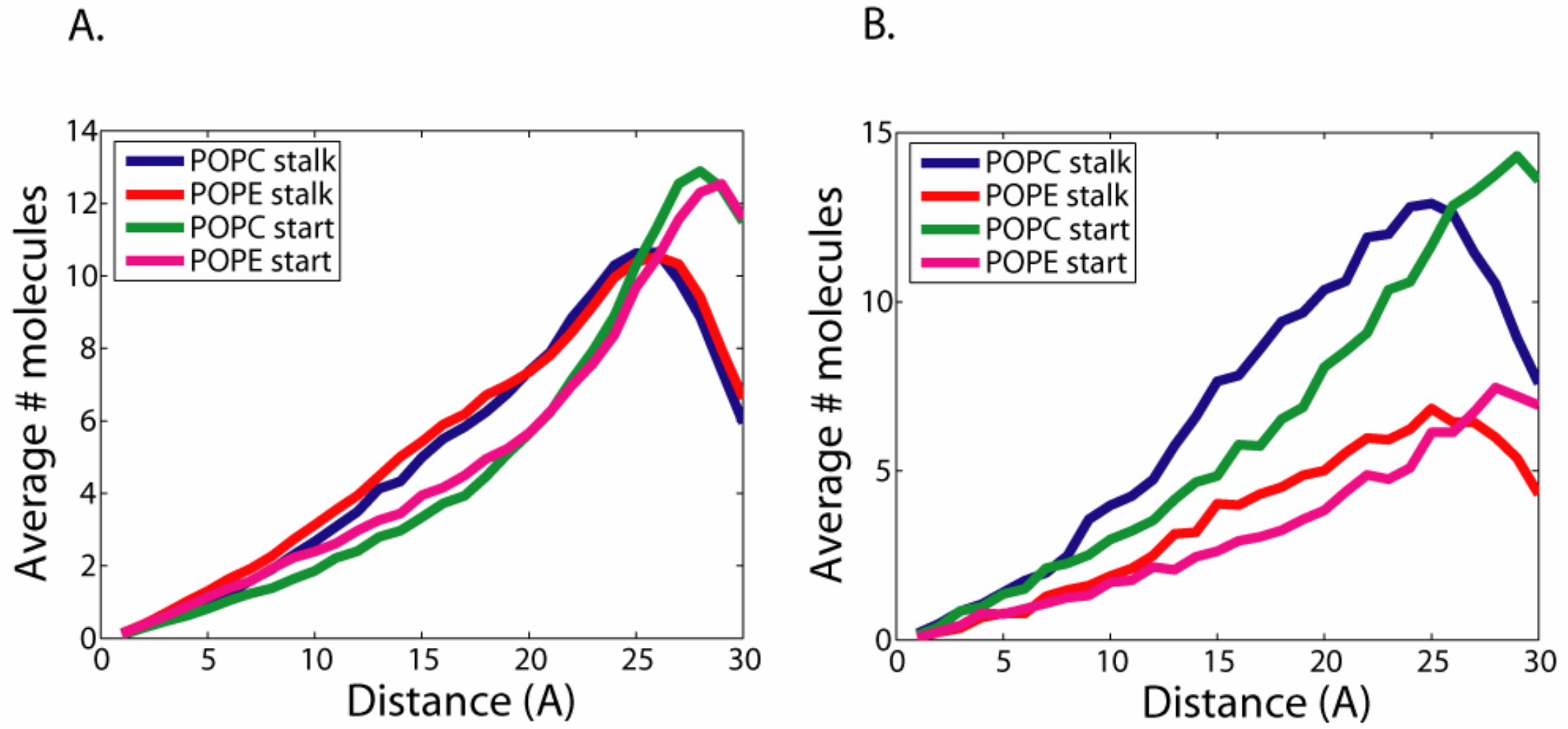

Figure S2. Radial distribution functions for POPC and POPE lipids. Plotted are average radial distributions relative to the stalk contact point for first trajectory snapshot after stalk formation for each simulation run calculated for 1:1 POPC:POPE (**A**) and 2:1 POPC:POPE (**B**) vesicles. Because the vesicle surface curvature results in a non-uniform radial distribution profile, distributions from the start state are plotted for reference. Distributions plotted represent the average values over all molecular dynamics trajectories. No relative enrichment of either POPC or POPE is observed at either lipid mixture.
