# Supplementary material for: Control of Membrane Fusion Mechanism by Lipid Composition: Predictions from Ensemble Molecular Dynamics
Source: PLoS Comput Biol. 2007 Nov 16;3(11):e220. doi: 10.1371/journal.pcbi.0030220 (PMC2077900; doi:10.1371/journal.pcbi.0030220)
Supplement: Supplementary file 4 [file pcbi.0030220.sg003.pdf]

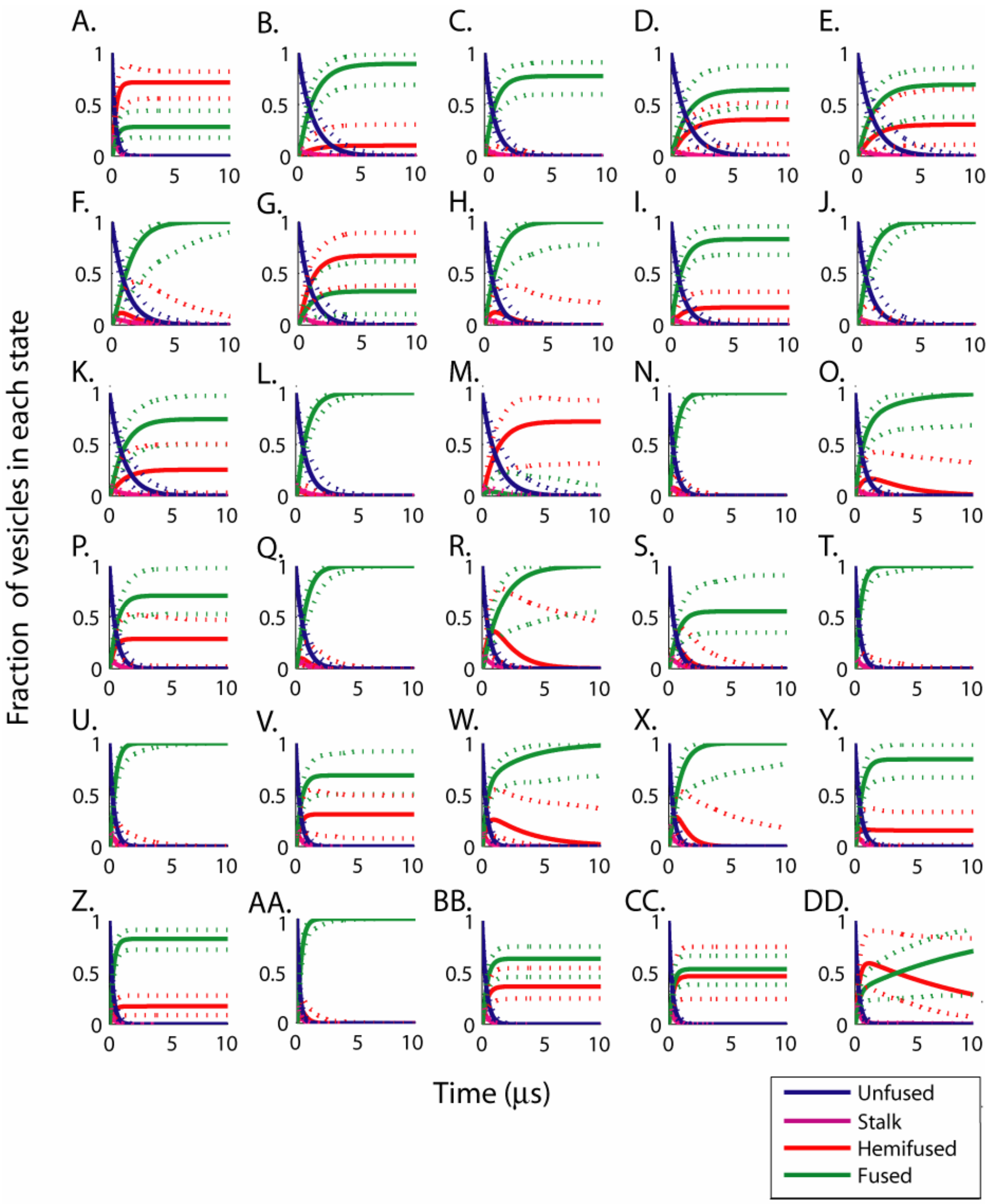

Figure S3. Estimated reaction kinetics for fusion at asymmetric lipid compositions differing between the inner and outer vesicle leaflets. Vesicle fusion was simulated at 30 lipid compositions ranging between approximately 50% and approximately 90% POPC content in the inner and outer leaflets. Each plot shows the estimated time-evolution of one such set of fusion reactions, with time plotted on the x axis and the fraction vesicles in each state plotted on the y axis. Vesicle compositions are marked **A-DD**, corresponding to the compositions listed in Table S2. Because long timescales were not sampled as efficiently in these simulations, compositions where the fusion reaction proceeds slowly have higher error in the long-timescale kinetics predicted.
