# Supplementary material for: Control of Membrane Fusion Mechanism by Lipid Composition: Predictions from Ensemble Molecular Dynamics
Source: PLoS Comput Biol. 2007 Nov 16;3(11):e220. doi: 10.1371/journal.pcbi.0030220 (PMC2077900; doi:10.1371/journal.pcbi.0030220)
Supplement: Supplementary file 5 [file pcbi.0030220.st001.pdf]

	Lipid Composition					
**Reaction**	**POPE**	90% CI	**POPC:POPE**	90% CI	**2:1 POPC:POPE**	90% CI
Unfused to Stalk	0*	-0.02 - 0.02	1.04	1.00 - 1.07	3.03	3.03 - 3.30
Stalk to Fused	1.85	1.81 - 1.88	0.49	0.44 - 0.54	0.18	0.06 - 0.29
Stalk to Hemifused1	0.46	0.45 - 0.48	0.96	0.89 - 1.04	1.53	1.17 - 2.00
Hemifused1 to Hemifused2	0.53	0.51 - 0.54	0.41	0.34 - 0.48	0.21	-.016 - 0.67
Hemifused2 to Fused	3.30	3.03 - 3.30	1.67	1.57 - 1.81	1.24	0.75 - 2.04

Table S1. Relative transition state energies. Energies are given in kcal/mol and calculated using forward rates obtained via MSM analysis of molecular dynamics trajectories at each lipid composition using the relation $\Delta\Delta G^{\ddagger} = - k_B T \ln k - \Delta G_0$, approximating each reaction as a single-barrier energy landscape with ΔG_0 set to the transition state free energy from unfused to stalk in pure POPE vesicles (*). 90% confidence intervals are generated via Dirichlet resampling of the MSM transition matrix. For reference, $k_B T = 0.645$ kcal/mol.
