# Supplementary material for: Control of Membrane Fusion Mechanism by Lipid Composition: Predictions from Ensemble Molecular Dynamics
Source: PLoS Comput Biol. 2007 Nov 16;3(11):e220. doi: 10.1371/journal.pcbi.0030220 (PMC2077900; doi:10.1371/journal.pcbi.0030220)
Supplement: Supplementary file 6 [file pcbi.0030220.st002.pdf]

	Inner PC:PE	Outer PC:PE	Total PC:PE	Stalk formation t½ (ns)	t½ 90% confidence interval		Estimated mean time to fusion (ns)
**A**	1.08	0.90	0.95	198.52	166.62	276.08	**
**B**	0.90	0.94	0.93	866.42	570.12	1269.81	1211.60
**C**	0.61	0.91	0.81	564.19	402.78	798.88	703.05
**D**	0.39	0.93	0.73	910.78	622.65	1358.56	905.40*
**E**	0.28	0.97	0.70	910.78	654.31	1573.16	978.07
**F**	0.09	0.96	0.59	823.19	600.39	1250.36	1573.91
**G**	1.06	0.86	0.91	696.41	494.28	1058.41	**
**H**	0.90	0.80	0.83	555.39	424.22	881.55	1198.04
**I**	0.65	0.82	0.77	572.87	410.19	792.69	761.46
**J**	0.37	0.84	0.67	662.62	508.10	1068.39	1111.64
**K**	0.29	0.82	0.62	814.22	560.73	1234.47	964.38
**L**	0.09	0.83	0.52	574.68	445.91	892.46	980.88
**M**	1.11	0.66	0.78	842.15	624.18	1490.39	**
**N**	1.01	0.69	0.77	340.41	269.15	477.96	669.06
**O**	0.61	0.63	0.63	443.08	326.65	604.77	1340.22
**P**	0.38	0.64	0.55	372.09	283.09	535.33	500.14*
**Q**	0.28	0.67	0.53	470.81	356.03	643.62	906.67
**R**	0.10	0.62	0.42	377.11	289.46	526.18	1679.11
**S**	1.16	0.51	0.65	382.03	299.64	499.47	444.94
**T**	1.05	0.49	0.62	276.21	214.97	360.09	522.42
**U**	0.63	0.50	0.54	263.67	213.96	355.87	531.04
**V**	0.40	0.51	0.48	257.08	195.33	334.16	343.68*
**W**	0.28	0.49	0.42	250.41	196.69	326.77	1351.35
**X**	0.10	0.50	0.35	262.53	205.66	352.20	947.67
**Y**	1.21	0.38	0.56	311.09	224.57	419.62	522.20
**Z**	0.99	0.40	0.53	197.05	160.81	256.84	304.69
**AA**	0.62	0.39	0.45	153.73	123.89	190.72	408.44
**BB**	0.41	0.40	0.40	248.08	204.19	313.21	294.56*
**CC**	0.29	0.38	0.35	203.33	162.78	256.11	**
**DD**	0.10	0.40	0.30	201.22	166.42	259.89	1800.91

Table S2. Asymmetric lipid composition and times of fusion. Inner and outer leaflet molar ratios of POPC:POPE are listed for the 30 vesicle compositions plotted in Figure 9. Labels **A-DD** correspond to labels in the figure. Also shown are $t_{½}$ values for reaction of unfused vesicles to form a stalk-like state and estimated mean times to full fusion for each composition. Mean times to fusion were estimated by calculating mean first passage times from the unfused to the fused state using a Markov State Model constructed for each reaction condition. As noted earlier, the uncertainty in these estimates is higher because they reflect long-timescale behavior that is less well sampled in the simulations. Values marked ** are too poorly sampled to estimate. Values marked * are estimated but with high uncertainty.
